# A Danish case of bleomycin‐induced flagellate erythema

**DOI:** 10.1002/ccr3.3220

**Published:** 2020-09-10

**Authors:** Christian A. S. Soerensen, Kristine Appel Uldall Pallesen

**Affiliations:** ^1^ Department of Dermatology Aarhus University Hospital Aarhus Denmark

**Keywords:** bleomycin, flagellate erythema, hyperpigmentation

## Abstract

Patients who are undergoing treatment with bleomycin and develop prurigo or rash should be suspected of bleomycin‐induced flagellate erythema and treated early with antihistamines, topical, and oral corticosteroids.

## Graphical abstract

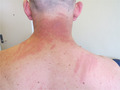

## 1 INTRODUCTION

Flagellate erythema is a rare side effect to treatment with bleomycin that may require discontinuation of the antineoplastic drug. Based on a case of bleomycin‐induced flagellate erythema, we give suggestions on how they manage the condition.

Flagellate erythema is characterized by pruritic linear infiltrated erythematous lesions, arranged in flagellate patterns. It is known to be associated with ingestion of shiitake mushrooms, dermatomyositis, adult‐onset Still's disease, and treatment with antineoplastic agents such as bleomycin.^1^ Although bleomycin‐induced flagellate erythema (BIFE) is a well‐described condition in the literature, it is rarely seen in clinical practice.^2^ Here, we present a case of BIFE in a male treated with bleomycin for testis cancer.

## 2 CASE REPORT

**FIGURE 1:**
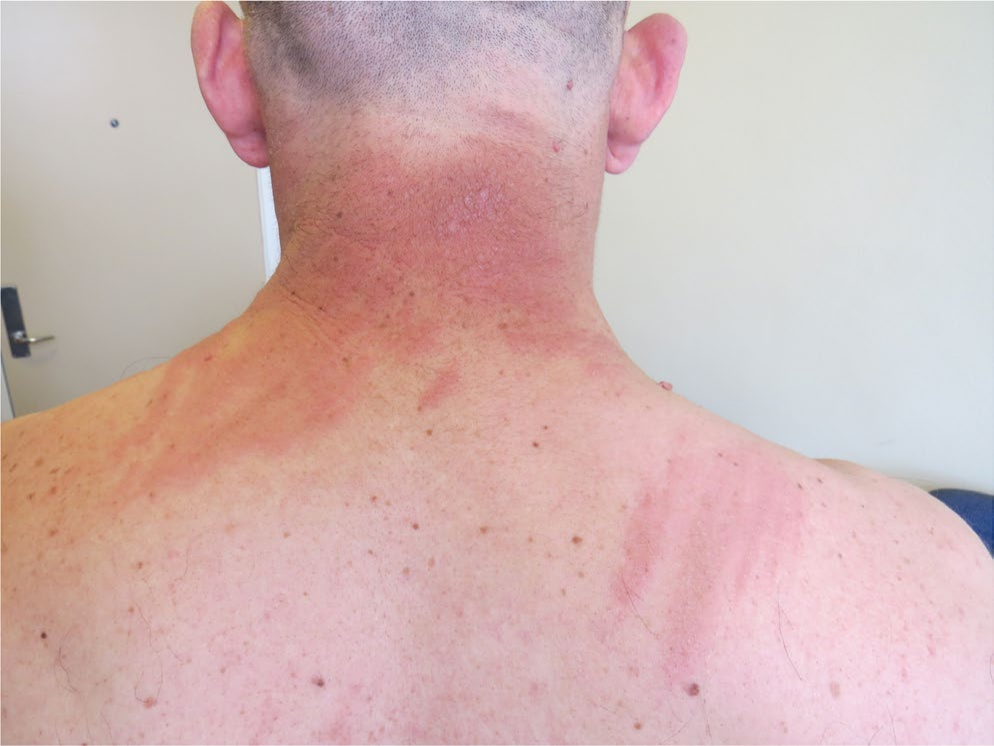
Multiple linear streaks of erythematous lesions with af “flagellate pattern” on the upper part of the back

A 49‐year‐old previously healthy man, diagnosed with nonseminoma testis cancer with retroperitoneal metastatic disease, was treated with unilateral orchiectomy, followed by three series of BEP (bleomycin, etoposide, and cisplatin) and pegfilgrastim. Shortly after the first series, the patient developed a pruritic sensation on the back of his neck and upper trunk. Within a few days, it progressed into a rash with the appearance of papules, plaques, and linear whip‐like formations of erythematous hyperpigmentation (Figure 1).

Based on the medical history and the clinical features of the rash, the patient was diagnosed with BIFE. Initially, the patient was treated with antihistamines and topical group III corticosteroids.. In addition 25 mg of prednisolone was added daily after the second series, but the rash and pruritus worsened and spread to other parts of the trunk and extremities as the antineoplastic regimen was continued for a total of three series. The patient reported that all three supportive treatments had moderate effects despite the aggravation of the rash and pruritus. The skin lesions and pruritus started to resolve weeks after the antineoplastic treatment was discontinued. At the 11‐month follow‐up, the patient reported total remission of the skin areas with hyperpigmentation, except for a minor area on the back of his neck.

## 3 DISCUSSION

Bleomycin‐induced flagellate erythema was first described by Moulin et al in 1970.^3^ It is seen in 8%‐20% of the treated patients and is reported to develop from 2 hours to several weeks after the initial bleomycin treatment. The skin manifestations are often self‐limiting.^4^ Bleomycin is inactivated by the enzyme bleomycin hydrolase that is widely distributed in normal tissues except for the lungs and skin, which makes these sites target of toxicity. BIFE is a cutaneous side effect to treatment with bleomycin among several others, including Raynaud's phenomenon, gangrene, fibrosis, alopecia, edema, nail changes, and neutrophilic eccrine hidradenitis (NEH).^5^ The development of the condition appears to be independent of the dose and mode of administration,^4^ and severe cases may require discontinuation of bleomycin, since no specific treatment has been identified.^1^, ^6^ However, as shown in the present case, it may be possible to continue the treatment with bleomycin with supportive therapy of antihistamines, topical, and oral corticosteroids.^7^, ^8^, ^9^ In most cases, complete remission of BIFE was reported months after discontinuation of bleomycin, while the present case and several others showed some degree of persistent hyperpigmentation up to 1 year after the treatment.^10^ Patients who are undergoing treatment with bleomycin and present symptoms of cutaneous prurigo or rash should be suspected of BIFE. Early treatment with antihistamines, topical, and oral corticosteroids should be considered to ameliorate the symptoms, with the goal that the patient may complete the intended antineoplastic regime. The patient should be included in the decision‐making of continuing the treatment based on the goal and expected length of the treatment, combined with the subjective symptoms, and the information of the self‐limiting nature of BIFE. Whether the risk of persistent hyperpigmentation should be part of the consideration is questionable, as the extent and distribution not formerly has been accurately evaluated. In addition, the present and former cases had a maximum of 1‐year follow‐up, why further improvement may occur.

## CONFLICT OF INTEREST

None declared.

## AUTHOR CONTRIBUTION

Both authors were involved in the clinical management of the patient and have read and approved the final manuscript. CS: wrote the manuscript and served as corresponding author. KP: revised and contributed to the manuscript.

## ETHICAL APPROVAL

This article does not contain any clinical studies with human patients performed by any of the authors.
